# A Hypertension-Associated tRNA^Ala^ Mutation Alters tRNA Metabolism and Mitochondrial Function

**DOI:** 10.1128/MCB.00199-16

**Published:** 2016-06-29

**Authors:** Pingping Jiang, Meng Wang, Ling Xue, Yun Xiao, Jialing Yu, Hui Wang, Juan Yao, Hao Liu, Yanyan Peng, Hanqing Liu, Haiying Li, Ye Chen, Min-Xin Guan

**Affiliations:** a Institute of Genetics, Zhejiang University, and Department of Genetics, Zhejiang University School of Medicine, Hangzhou, Zhejiang, China; b Attardi Institute of Mitochondrial Biomedicine, Wenzhou Medical University, Wenzhou, Zhejiang, China; c Department of Cardiology, The First Affiliated Hospital, Wenzhou Medical University, Wenzhou, Zhejiang, China; d Collaborative Innovation Center for Diagnosis and Treatment of Infectious Diseases, Zhejiang University, Hangzhou, China; e Joint Institute of Genetics and Genomic Medicine between Zhejiang University and University of Toronto, Zhejiang University, Hangzhou, Zhejiang, China

## Abstract

In this report, we investigated the pathophysiology of a novel hypertension-associated mitochondrial tRNA^Ala^ 5655A → G (m.5655A → G) mutation. The destabilization of a highly conserved base pairing (A1-U72) at the aminoacyl acceptor stem by an m.5655A → G mutation altered the tRNA^Ala^ function. An *in vitro* processing analysis showed that the m.5655A → G mutation reduced the efficiency of tRNA^Ala^ precursor 5′ end cleavage catalyzed by RNase P. By using cybrids constructed by transferring mitochondria from lymphoblastoid cell lines derived from a Chinese family into mitochondrial DNA (mtDNA)-less (ρ^o^) cells, we showed a 41% reduction in the steady-state level of tRNA^Ala^ in mutant cybrids. The mutation caused an improperly aminoacylated tRNA^Ala^, as suggested by aberrantly aminoacylated tRNA^Ala^ and slower electrophoretic mobility of mutated tRNA. A failure in tRNA^Ala^ metabolism contributed to variable reductions in six mtDNA-encoded polypeptides in mutant cells, ranging from 21% to 37.5%, with an average of a 29.1% reduction, compared to levels of the controls. The impaired translation caused reduced activities of mitochondrial respiration chains. Furthermore, marked decreases in the levels of mitochondrial ATP and membrane potential were observed in mutant cells. These caused increases in the production of reactive oxygen species in the mutant cybrids. The data provide evidence for the association of the tRNA^Ala^ 5655A → G mutation with hypertension.

## INTRODUCTION

Hypertension is a major global public health problem, affecting approximately 1 billion people worldwide, including 265 million adults in China (1, 2). Hypertension as a polygenic, multifactorial, and highly heterogeneous disorder could be caused by single-gene or multifactorial conditions resulting from interactions between environmental and inherited risk factors (3). In particular, mitochondria can regulate various aspects of vascular function, thereby being critical for the pathogenesis of hypertension (4, 5). The maternal transmission of hypertension reported in several studies further supports mitochondrial involvement in hypertension (5, 6). The human mitochondrial genome (mitochondrial DNA, or mtDNA) encodes 13 subunits of the oxidative phosphorylation system, 2 rRNAs, and 22 tRNAs required for mitochondrial protein synthesis (7). Among these tRNAs, 8 tRNAs, such as tRNA^Glu^ and tRNA^A1a^, reside on the cytosine-rich light (L) strand; the remaining tRNAs, including tRNA^Lys^ and tRNA^His^, are located on the guanine-rich heavy (H) strand (8, 9). Mitochondrial tRNA genes were proposed to be the hot spots for mutations associated with cardiovascular disorders, including hypertension (10–12). These hypertension-associated tRNA mutations were the tRNA^Ile^ 4263A → G and 4295A → G mutations and the tRNA^Met^ 4435A → G and 4401A → G mutations in the junction of the tRNA^Met^ and tRNA^Gln^ genes (13–16). These mutations have structural and functional consequences, including the processing of RNA precursors, nucleotide modification, and aminoacylation (17, 18). The m.4263A → G and m.4401A → G (where “m” indicates mitochondrial sequence) mutations altered the processing of corresponding tRNA precursors, catalyzed by RNase P (13, 15, 19), while the m.4295A → G and m.4435A → G mutations may affect the nucleotide modification at position 37, at the 3′ end adjacent to this position of the tRNA^Ile^ and tRNA^Met^ (14, 16, 20, 21). However, the pathophysiology of these tRNA mutations remains poorly understood. Thus, it is necessary to establish the link between hypertension and mitochondrial dysfunction and their cause/effect relation.

**FIG 1:**
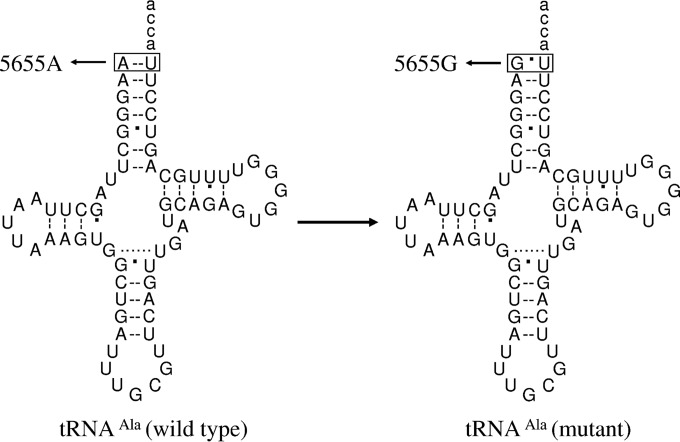
Cloverleaf structure of human mitochondrial tRNA^Ala^. An arrow denotes the location of the m.5655A → G mutation.

As part of a genetic screening program for hypertension in a cohort of 2,070 Han Chinese hypertensive subjects, we identified the T-to-C transition at position 5655 (5655A → G) at the 5′ end of the tRNA^Ala^ gene in three genetically unrelated probands whose families exhibited a maternal transmission of hypertension (see the supplemental material). As shown in Fig. 1, the m.5655A → G mutation was located at the processing site for the tRNA 5′ end precursors, catalyzed by RNase P (19, 22). Furthermore, the m.5655A → G mutation changes the highly conserved base pairing (A1-U72) at the aminoacyl acceptor stem to G1-U72. It was hypothesized that the destabilization of base pairing (1A-72U) and change of the processing site for the tRNA 5′ end precursor by the m.5655A → G mutation altered the structure and function of tRNA^Ala^. In particular, the mutation may affect the aminoacylation capacity and stability of this tRNA. A failure in tRNA metabolism leads to the impairment of mitochondrial translation and respiration (17, 18, 23). It was also proposed that mitochondrial dysfunctions caused by the tRNA mutation alter the production of ATP and reactive oxygen species (ROS). To investigate the pathogenic mechanism of the m.5655A → G mutation in these Chinese families, cybrid cell lines were constructed by transferring mitochondria from lymphoblastoid cell lines derived from an affected matrilineal relative carrying the m.5655A → G mutation and from a control individual lacking the mtDNA mutation into human mtDNA-less [*rho*^0^] cells (24, 25). These cybrid cell lines were first examined for the presence and degree of the mtDNA mutation. These cell lines were then assessed for the effects of the mtDNA mutation on tRNA metabolism, including aminoacylation efficiency and steady-state levels, mitochondrial translation, respiration, and production of ATP and ROS.

## MATERIALS AND METHODS

### Subjects.

A cohort of 2,070 Chinese hypertensive probands were recruited from the Hypertension Clinic, Wenzhou Medical University, China, as detailed previously (26, 27). This study was in compliance with the Declaration of Helsinki. Informed consent, blood samples, and clinical evaluations were obtained from all participating family members under protocols approved by the Ethics Committees of Zhejiang University and the Wenzhou Medical University. Diagnosis of hypertension was based on the criteria of the American Heart Association (28). All available members of three pedigrees carrying the m.5655A → G mutation were evaluated at length to identify both personal and family medical histories of hypertension and other clinical abnormalities, as detailed elsewhere (15, 26, 27). The 800 Chinese control subjects were obtained from a panel of unaffected subjects from Han Chinese ancestry from the same region.

### Mutational analysis of mitochondrial DNAs.

Genomic DNA was isolated from whole blood of 2,070 hypertensive probands and 800 Chinese control subjects by using a QIAamp DNA blood minikit (no. 51104; Qiagen). Subjects' DNA fragments spanning the tRNA^Ala^ gene were amplified by PCR using oligodeoxynucleotides corresponding to mtDNA at positions 5258 to 6031 (7). Each fragment was purified and subsequently analyzed by direct sequencing. These sequence results were compared with the updated consensus Cambridge sequence (GenBank accession number NC_012920) (7). The entire mtDNAs of three probands, WHP20-II-13, WHP21-II-3, and WHP22-II-5, and of a Chinese control subject (C1) were PCR amplified in 24 overlapping fragments using sets of oligonucleotide primers, as described previously (29). These sequence results were compared with the updated consensus Cambridge sequence, as described above. To quantify the m.5655A → G mutation, the PCR segment (280 bp) was amplified using genomic DNA as the template and oligodeoxynucleotides corresponding to mtDNA at positions 5536 to 5816 and subsequently digested with a restriction enzyme, MnlI. The 5655A → G mutation created the site for this restriction enzyme. Equal amounts of various digested samples were then analyzed by electrophoresis through 7% polyacrylamide gels. The proportions of digested and undigested PCR product were determined by the ImageQuant program after ethidium bromide staining to determine if the m.5655A → G mutation was in homoplasmy in these subjects.

### Cell lines and culture conditions.

Subjects and mutational analysis of mitochondrial DNA were as detailed in the supplemental material. Lymphoblastoid cell lines were immortalized by transformation with the Epstein-Barr virus, as described previously (30). Cell lines derived from the proband WHP20-II-13 carrying the m.5655A → G mutation and from one Han Chinese control, C1, belonging to the same mtDNA haplogroup but lacking this mutation were used for the generation of cybrid cell lines grown in RPMI 1640 medium (Invitrogen) supplemented with 10% fetal bovine serum (FBS). The bromodeoxyuridine (BrdU)-resistant 143B.TK^−^ cell line was grown in Dulbecco's modified Eagle medium (DMEM) (Life Technologies) (containing 4.5 mg of glucose and 0.11 mg pyruvate/ml), supplemented with 100 μg of BrdU/ml and 5% FBS. Transformation by cytoplasts of mtDNA-less ρ°206 cells was performed as described elsewhere (24, 25). The mtDNA-less ρ°206 cell line, derived from 143B.TK^−^ cells, was grown under the same conditions as the parental line, except for the addition of 50 μg of uridine/ml. The presence and level of the m.5655A → G mutation and mtDNA copy numbers in cybrid cell lines were analyzed as detailed in the supplemental material (25).

### Mitochondrial RNase P assay.

The tRNA^Ala^ wild-type and mutant precursors corresponding to mtDNA at positions 5567 to 5692 of human mtDNA were cloned into in the TA vector (Invitrogen). The labeled RNA substrates (125 nucleotides [nt]) were transcribed with T7 RNA polymerase and were purified by denaturing polyacrylamide gel electrophoresis. Processing assays were carried out in parallel for wild-type and mutant substrates as detailed previously (15). Enzyme reaction mixtures were incubated at 21°C. After 4, 8, 12, 16, and 30 min, aliquots were withdrawn, and the reaction was stopped by addition of an equal volume of denaturing gel loading buffer (85% formamide, 10 mM EDTA). Reaction products were resolved via denaturing polyacrylamide gel electrophoresis and detected by phosphor storage autoradiography. ImageQuant TL (GE Healthcare) was used for relative quantification of reaction products.

### Mitochondrial tRNA Northern analysis.

Total mitochondrial RNAs (mtRNAs) were obtained from mitochondria isolated from mutant and control cell lines (∼2.0 × 10^8^ cells) using a ToTALLY RNA kit (Ambion), as described previously (31). Two micrograms of total mitochondrial RNA was electrophoresed through a 10% polyacrylamide–7 M urea gel in Tris-borate-EDTA (TBE) buffer (after heating the sample at 65°C for 10 min) and then electroblotted onto a positively charged nylon membrane (Roche) for hybridization analysis with oligodeoxynucleotide probes. For the detection of tRNA^Ala^, tRNA^Lys^, tRNA^His^, and tRNA^Glu^, nonradioactive digoxigenin (DIG)-labeled oligodeoxynucleotides specific to each RNA and 5S RNA were as detailed previously (9, 15, 32–34). DIG-labeled oligodeoxynucleotides were generated by using a DIG oligonucleotide tailing kit (Roche). The hybridization was carried out as detailed elsewhere (9, 15, 32–34). Quantification of density in each band was done as detailed previously (9, 15, 32–34).

### Mitochondrial tRNA aminoacylation analysis.

Total mitochondrial RNAs were isolated under acid conditions (34–36). Two micrograms of total mitochondrial RNAs was electrophoresed at 4°C through an acid (pH 5.2) 10% polyacrylamide–7 M urea gel to separate the charged and uncharged tRNAs as detailed elsewhere (34, 35). The gels were then electroblotted onto a positively charged nylon membrane (Roche) for the hybridization analysis with DIG-labeled oligodeoxynucleotide probes for tRNA^Ala^, tRNA^Thr^, tRNA^Ser(AGY)^, and tRNA^Gly^ as detailed previously (9, 15, 32–36). To further distinguish nonaminoacylated tRNA from aminoacylated tRNA, samples of tRNAs were deacylated by being heated for 10 min at 60°C at pH 8.3 and then run in parallel (35, 36). DIG-labeled oligodeoxynucleotides and quantification of density in each band were generated as described above.

### Western blot analysis.

Western blotting was performed as detailed previously (32, 34). The antibodies used for this investigation were the following: TOM20 (ab56783), ND1 (ab74257), ATP6 (ab101908), and CO2 (subunit II of cytochrome *c* oxidase) (ab110258) from Abcam; ND4 (sc-20499-R) and ND6 (sc-20667) from Santa Cruz Biotechnology; and CYTB (apocytochrome *b*) (55090-1-AP) from Proteintech. Peroxidase Affinipure goat anti-mouse IgG and goat anti-rabbit IgG (Jackson) were used as secondary antibodies, and protein signals were detected using an enhanced chemiluminescence (ECL) system (CWbio). Quantification of density in each band was performed as detailed previously (32, 34).

### Measurements of oxygen consumption.

The rates of oxygen consumption in cybrid cell lines were measured with a Seahorse Bioscience XF-96 extracellular flux analyzer (Seahorse Bioscience), as detailed previously (32, 37).

### Enzymatic assays.

The enzymatic activities of complexes I (NADH ubiquinone oxidoreductase), II (cytochrome *c*), III (ubiquinone cytochrome *c* oxidoreductase), and IV (cytochrome *c* oxidase) were assayed as detailed elsewhere (34, 38, 39).

### ATP measurements.

A CellTiter-Glo luminescent cell viability assay kit (Promega) was used for the measurement of cellular and mitochondrial ATP levels, according to the modified manufacturer's instructions (32, 39).

### Assessment of mitochondrial membrane potential.

Mitochondrial membrane potential (ΔΨm) was assessed with a JC-10 assay kit (microplate; Abcam) according to the manufacturer's recommendations with some modifications, as detailed elsewhere (32).

### Measurement of ROS production.

Measurement of total cellular ROS generation was performed as detailed elsewhere (32, 40, 41). The level of ROS generation by mitochondria in living cells was analyzed using the mitochondrial superoxide indicator MitoSOX-Red (Invitrogen), as detailed previously (37, 38).

### Computer analysis.

Statistical analysis was carried out using the unpaired, two-tailed Student *t* test of the Microsoft Excel program or Excel for Macintosh (version 2007). Differences were considered significant at a *P* value of <0.05.

## RESULTS

### Identification of the tRNA^Ala^ 5655A → G mutation.

The m.5655A → G mutation in the tRNA^Ala^ gene was found in three genetically unrelated probands in a cohort of 2,070 Chinese hypertensive subjects but was absent in 800 Chinese control subjects. As shown in Fig. 1, the m.5655A → G mutation was located at the 5′ end of tRNA, which is the processing site for the tRNA^Ala^ 5′ end precursors of the light strand, catalyzed by RNase P (19, 22), and altered the A-U base pairing (A1-U72) at the aminoacyl acceptor stem of tRNA^Ala^. The sequence analysis of the entire mtDNA in these probands exhibited the identical m.5655A → G mutations and distinct sets of polymorphisms belonging to the Eastern Asian haplogroups B4 and D4 (see Table S1 in the supplemental material) (42). However, there were no other functional significant variants in their mitochondrial genomes.

### Clinical and genetic evaluation of three Chinese families.

Members of three Chinese families carrying the m.5655A → G mutation underwent a physical examination, laboratory assessment of cardiovascular disease risk factors, and routine electrocardiography. In the pedigree WHP20, 12 of 19 matrilineal relatives had a wide range in levels of severity of hypertension (with blood pressure greater than 140/90 mm Hg even with treatment for hypertension), while only 4 of 28 nonmaternal relatives suffered from hypertension. None of the offspring of five affected fathers exhibited hypertension, while one affected subject, IV-2, was the son of subject III-1, who married an affected wife (III-2). As shown in Table S2 in the supplemental material, the age at onset of hypertension in the maternal kindred varied from 38 years to 80 years, with an average of 56.7 years. Furthermore, 3 of 9 matrilineal relatives in the pedigree WHP21 suffered from hypertension, while 9 of 17 matrilineal relatives in the pedigree WHP22 exhibited hypertension. None of the other members in these families had hypertension. The average ages at onset of hypertension in these families were 50.3 and 56.4 years, respectively. There was no evidence that any member of these families had any other cause to account for hypertension. However, no other clinical abnormalities were observed in the maternal kindred. Further analysis showed that the m.5655A → G mutation was present in homoplasmy in all matrilineal relatives but not in other members of these families (data not shown).

### Generation of cybrid cell lines.

The lymphoblastoid cell lines derived from one affected subject (WHP20-II-13) carrying the m.5655A → G mutation and one control individual (C1) lacking this mtDNA mutation but belonging to the same mtDNA haplogroup were enucleated and subsequently fused to a large excess of mtDNA-less human ρ^o^206 cells, derived from the 143B.TK^−^ cell line (24, 25). The cybrid clones were isolated by growing the fusion mixtures in selective DMEM containing BrdU and lacking uridine (24). Between 25 and 45 days after fusion, 10 to 15 presumptive mitochondrial cybrids derived from each donor cell line were isolated and subsequently analyzed for the presence and level of the m.5655A → G mutation and copy number of mtDNA, respectively. The results confirmed the absence of the mtDNA mutation in the control clones and its presence in homoplasmy in all cybrids derived from the mutant cell line (data not shown). Furthermore, the karyotypes for each cybrid line were examined. Three cybrids derived from each donor cell line with similar mtDNA copy numbers and the same karyotype as 143B cell lines were used for the biochemical characterization described below.

### Impairment in the 5′ end processing of the mitochondrial tRNA^Ala^ precursor.

**FIG 2:**
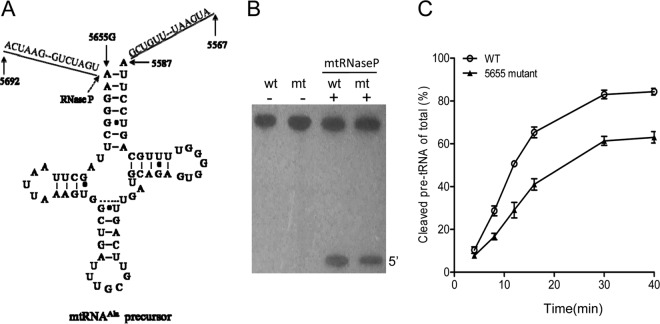
*In vitro* assay for the processing of mitochondrial tRNA^Ala^ precursor. (A) Human mitochondrial tRNA^Ala^ precursors. Thirty-seven nucleotides (nt) of 5′ end leader and 20 nt of the 3′ end trailer are shown, including the 5655A → G substitution. (B) *In vitro* processing assays. Processing assays with mitochondrial RNase P were carried out in parallel for wild-type (wt) and mutant (mt) substrates. Reaction products were resolved by denaturing polyacrylamide gel electrophoresis and detected by phosphor storage autoradiography. (C) Quantification of the processing efficiency of tRNA^Ala^ precursor. The ImageQuant program was used for relative quantification of reaction products. The relative processing efficiency was calculated from the initial phase of the reaction. The calculations were based on three independent determinations.

We employed an *in vitro* processing system to determine if the primary defect arising from the m.5655A → G mutation is the perturbed processing of the tRNA's 5′ end by RNase P. The wild-type and mutant tRNA^Ala^ precursors corresponding to mtDNA at positions 5692 to 5567 (Fig. 2A) were prepared by *in vitro* transcription. The *in vitro* processing kinetics of the wild-type and mutant substrates were determined as detailed elsewhere (15). No qualitative processing alteration of the mutant tRNA^Ala^ precursor was observed, but the processing efficiency of the mutant tRNA^Ala^ transcript was reduced compared with that of the wild-type tRNA^Ala^ transcript (Fig. 2B). In particular, the processing efficiency of the mutant tRNA^Ala^ transcript was ∼65% of that of the wild-type tRNA^Ala^ transcript (Fig. 2C).

### Marked decrease in the steady-state level of mitochondrial tRNA^Ala^.

**FIG 3:**
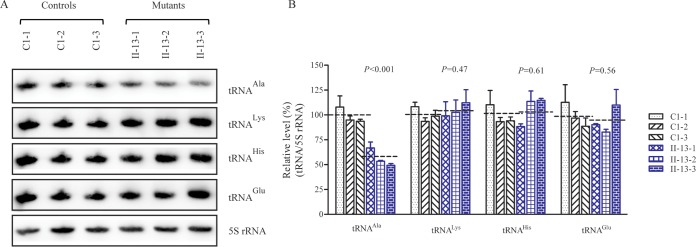
Northern blot analysis of mitochondrial tRNA. (A) Two micrograms of total mtRNA samples from the various cell lines was electrophoresed through a denaturing polyacrylamide gel, electroblotted, and hybridized with DIG-labeled oligonucleotide probes specific for the tRNA^Ala^, tRNA^Lys^, tRNA^His^, tRNA^Glu^, and 5S RNA. (B) Quantification of the tRNA levels. Average relative tRNA^Ala^, tRNA^Lys^, tRNA^His^, and tRNA^Glu^ content per cell was normalized to the average content per cell of 5S RNA in three cybrid cell lines derived from one affected subject (WHP20-II-13) carrying the m.5655A → G mutation and in three cybrid cell lines derived from one Chinese control subject (C1). The values are expressed as percentages of the average values for the control cell line. The calculations were based on three independent determinations in each cell line. The error bars indicate two standard errors of the means. *P* indicates the significance, according to the Student *t* test, of the differences between the mean of three mutant cybrids and mean of three control cybrids.

To examine whether the m.5655A → G mutation alters the tRNA metabolism, total mitochondrial RNA from cybrid cell lines was subjected to Northern blotting and hybridized with DIG-labeled oligodeoxynucleotide probes specific for tRNA^Ala^ and other tRNAs. The other probes were specific for tRNA^Lys^ and tRNA^His^ as representatives of the whole heavy (H)-strand transcription unit and for tRNA^Glu^ derived from the light (L)-strand transcription unit (8, 9). The amounts of tRNA^Ala^ in mutant cells were markedly decreased compared to those in control cells (Fig. 3A). For comparison, the average level of each tRNA in the control or mutant cell line was normalized to the average level in the same cell line for the reference 5S RNA. The average steady-state level of tRNA^Ala^ in the three mutant cell lines was 56.5% (*P* = 0.01) of the level of the three control cell lines after normalization to 5S RNA. However, the average steady-state levels of tRNA^Lys^, tRNA^His^, and tRNA^Glu^ in the three mutant cell lines were 104.6%, 103.2%, and 91.9% of those in the three control cell lines, respectively (Fig. 3B).

### Altered aminoacylation of tRNA^Ala^.

**FIG 4:**
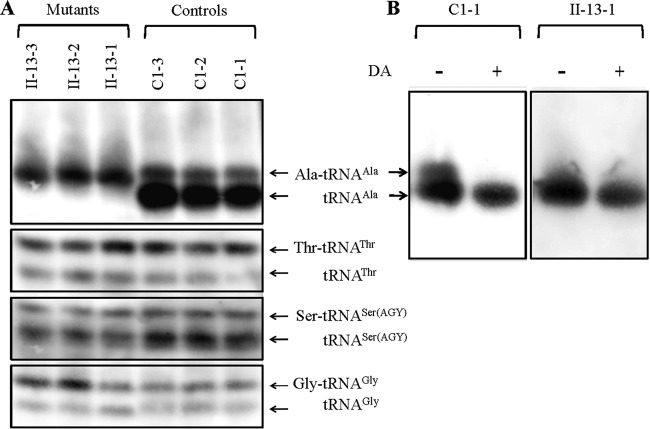
*In vivo* aminoacylation assays. (A) Two micrograms of total mitochondrial RNA purified from six cell lines (the same as shown in Fig. 3A) under acid conditions was electrophoresed at 4°C through an acid (pH 5.2) 10% polyacrylamide–7 M urea gel, electroblotted, and hybridized with a DIG-labeled oligonucleotide probe specific for the tRNA^Ala^. The blots were then stripped and rehybridized with tRNA^Thr^, tRNA^Ser(AGY)^, and tRNA^Gly^. (B) The samples from one control (C1-1) and one mutant (II-13-1) cell line were deacylated (DA) by heating for 10 min at 60°C at pH 8.3 and electrophoresed as described above. Aminoacylation assays for tRNA^Ala^ were carried out in parallel for aminoacylated and deacylated samples.

The aminoacylation capacities of tRNA^Ala^, tRNA^Thr^, tRNA^Ser(AGY)^, and tRNA^Gly^ in control and mutant cell lines were examined by the use of electrophoresis in an acid polyacrylamide-urea gel system to separate uncharged tRNA species from the corresponding charged tRNA, electroblotting, and hybridizing with the above tRNA probes. As shown in Fig. 4A, there were two bands for charged (upper band) and uncharged (lower band) species of wild-type tRNA^Ala^, while only one band occurred in the mutated tRNA^Ala^, which migrated more slowly than that of the wild-type tRNA^Ala^. To further distinguish nonaminoacylated tRNA from aminoacylated tRNA, samples of tRNAs were deacylated by being heated for 10 min at 60°C at pH 8.3 and then run in parallel. As shown in Fig. 4B, only one band (uncharged tRNA) was present in both the mutant and control cell lines after deacylation. These data indicated that the m.5655A → G mutation caused the aberrant metabolism of aminoacylated tRNA^Ala^. However, there were no obvious differences in electrophoretic mobility and aminoacylated efficiencies of other tRNAs between the mutant cell lines carrying the m.5655A → G mutation and the control cell lines lacking this mutation.

### Reduction in the level of mitochondrial proteins.

**FIG 5:**
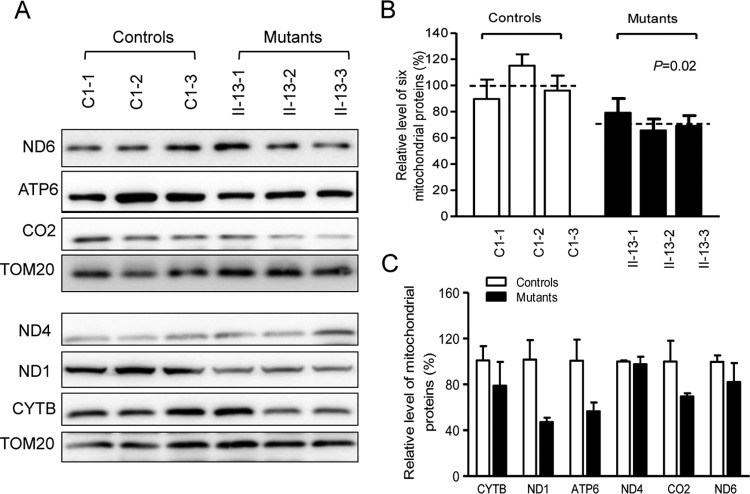
Western blot analysis of mitochondrial proteins. (A) Twenty micrograms of total cellular proteins from various cell lines was electrophoresed through a denaturing polyacrylamide gel, electroblotted, and hybridized with seven respiratory complex subunits in mutant and control cells with TOM20 as a loading control. CO2, subunit II of cytochrome *c* oxidase; ND1, ND4, ND5, and ND6, subunits 1, 4, 5, and 6 of the reduced nicotinamide-adenine dinucleotide dehydrogenase; ATP6, subunit 6 of the H^+^-ATPase; CYTB, apocytochrome *b*. (B) Quantification of mitochondrial protein levels. The levels of mitochondrial proteins in three mutant cell lines and three control cell lines were determined as described elsewhere (28). (C) Quantification of seven respiratory complex subunits. The levels of CO2, ND1, ND4, ND5, ND6, ATP6, and CYTB in three mutant cell lines and three control cell lines were determined as described elsewhere (32). Graph details and symbols are explained in the legend of Fig. 3.

Western blot analysis was carried out to examine the steady-state levels of six respiratory complex subunits (encoded by mtDNA) in mutant and control cells with TOM20 (mitochondrial protein encoded by the nuclear gene) as a loading control. The overall levels of six mitochondrial translation products in the mutant cell lines ranged from 21% to 37.5%, with an average of 29.1% (*P* = 0.02), relative to the mean value in the control cell lines. As shown in Fig. 5 and Table 1, mutant cell lines carrying the m.5655A → G mutation exhibited marked reductions (44% to 53%) in the levels of two polypeptides (ND1 and ATP6) harboring 8.4% to 8.5% alanine codons and relatively mild reductions (3% to 30%) in the levels of CO2, ND4, ND6, and CYTB carrying 4.6% to 6.6% alanine codons. However, the polypeptide levels in mutant cell lines, relative to those in control cell lines, did not significantly correlate with either the proportion or content of alanine codons, in contrast with what was previously shown in cells carrying the m.7445T → C mutation in the precursor of tRNA^Ser(UCN)^ gene or m.8344A → G mutation in the tRNA^Lys^ gene (9, 36).

**TABLE 1:** Usage of alanine codons in human mitochondrial genes and relative polypeptide levels in mutant cell lines

Polypeptide	No. of amino acids	No. of alanine residues	Alanine codon density (%)	Avg polypeptide level in mutants (% of controls)^*a*^
ND1	318	27	8.5	47.2
ND4	459	26	5.7	97.6
CO2	227	14	6.1	69.4
ND6	174	8	4.6	82.1
CYTB	378	25	6.6	78.9
ATP6	226	19	8.4	56.5
CO1	513	40	7.8	
ND5	603	44	7.3	
CO3	260	15	5.7	
ND2	347	20	5.8	
ND3	115	8	1	
ND4L	98	9	1	
ATP8	68	0	0	

a Values are the average levels of the individual polypeptide in the mutant cell lines relative to levels in the control cell lines.

### Respiration defects in mutant cells.

**FIG 6:**
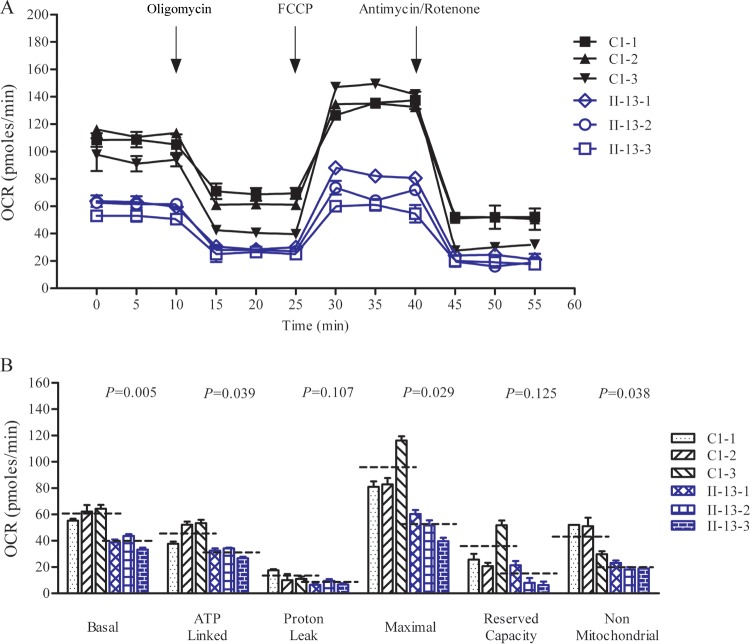
Respiration assays. (A) An analysis of O_2_ consumption in the various cell lines using different inhibitors. The rates of O_2_ (OCR) were first measured on 2 × 10^4^ cells of each cell line under basal conditions and then oligomycin (1.5 μM), carbonyl cyanide-*p*-(trifluoromethoxy)phenylhydrazone (FCCP) (0.5 μM), rotenone (1 μM), and antimycin A (1 μM) were sequentially added at the indicated times to determine different parameters of mitochondrial functions. (B) Graphs present the ATP-linked OCR, proton leak OCR, maximal OCR, reserve capacity, and nonmitochondrial OCR in mutant and control cell lines. Nonmitochondrial OCR was determined as the OCR after rotenone and antimycin A treatment. Basal OCR was determined as the OCR before oligomycin minus the OCR after rotenone and antimycin A. ATP-linked OCR was determined as the OCR before oligomycin minus the OCR after oligomycin. Proton leak was determined as the basal OCR minus the ATP-linked OCR. Maximal OCR was determined as the OCR after FCCP treatment minus the nonmitochondrial OCR. Reserve capacity was defined as the difference between maximal OCR after FCCP minus the basal OCR. The average of four determinations for each cell line is shown; the dashed lines represent the average values for each group. Graph details and symbols are explained in the legend to Fig. 3.

To evaluate if the m.5655A → G mutation alters cellular bioenergetics, we examined the oxygen consumption rates (OCR) of mutant and control cell lines. The basal OCR in the mutant cell lines was 64% (*P* = 0.005) relative to the mean value measured in the control cell lines (Fig. 6). The average values of ATP-linked OCR, proton leak OCR, maximal OCR, reserve capacity, and nonmitochondrial OCR in mutant cell lines were ∼65%, 58%, 54%, 32%, and 45%, respectively, relative to the mean values measured in the control cell lines (*P* = 0.039, 0.107, 0.029, 0.125, and 0.038, respectively) (Fig. 6).

**FIG 7:**
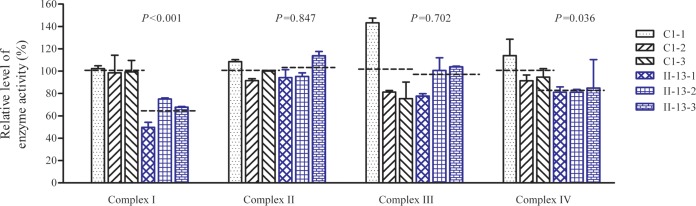
Enzymatic activities of respiratory chain complexes. The activities of respiratory complexes were investigated by enzymatic assay on complexes I, II, III, and IV in isolated mitochondrial membranes from three mutant and three control cybrid cell lines. The calculations were based on three independent determinations. Graph details and symbols are explained in the legend to Fig. 3.

The activities of respiratory complexes were also measured by isolating mitochondria from three mutant and three control cell lines. Complex I (NADH ubiquinone oxidoreductase) activity was determined by following the oxidation of NADH with ubiquinone as the electron acceptor. Complex III (ubiquinone cytochrome *c* oxidoreductase) activity was measured as a reduction of cytochrome *c* (III) using d-ubiquinol 2 as the electron donor. Complex IV (cytochrome *c* oxidase) activity was monitored by following the oxidation of cytochrome *c* (II). As shown in Fig. 7, the activities of complexes I, II, III, and IV in the three mutant cell lines were 64%, 101%, 94%, and 82%, respectively, relative to the mean values measured in the control cell lines (*P* < 0.001, 0.847, 0.702, and 0.036, respectively).

### Reduced levels in mitochondrial ATP production.

**FIG 8:**
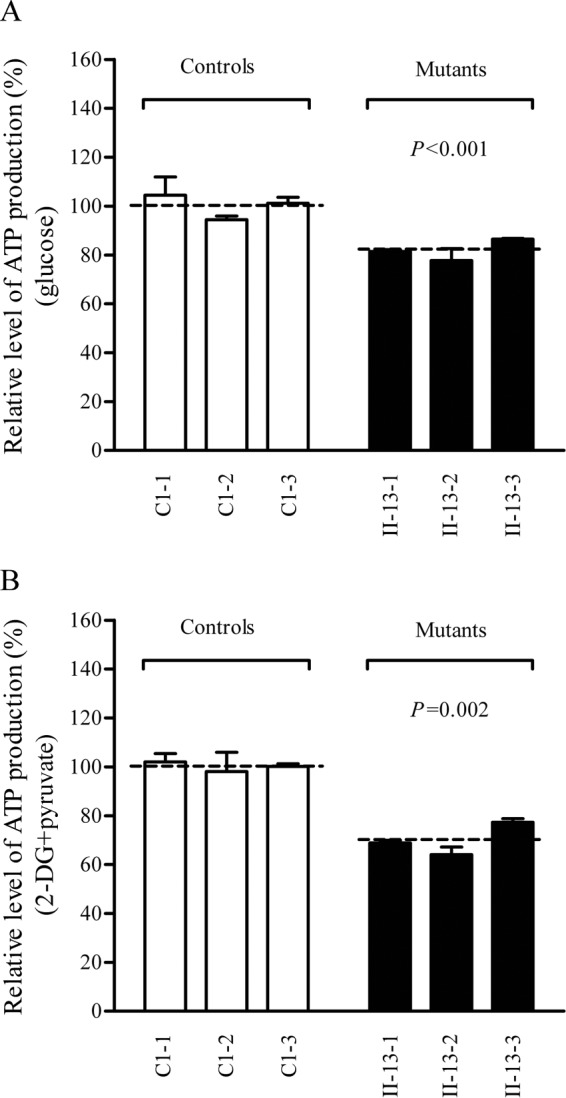
Measurement of cellular and mitochondrial ATP levels. Cells were incubated with 10 mM glucose or 5 mM 2-deoxy-d-glucose (2-DG) plus 5 mM pyruvate to determine ATP generation under mitochondrial ATP synthesis. Relative average levels per cell line of ATP in total cells (A) and in mitochondria (B) are shown. Six or seven determinations were made for each cell line. Graph details and symbols are explained in the legend to Fig. 3.

The capacity of oxidative phosphorylation in mutant and wild-type cells was examined by measuring the levels of cellular and mitochondrial ATP using a luciferin/luciferase assay. Populations of cells were incubated in the medium in the presence of glucose and 2-deoxy-d-glucose with pyruvate (32). As shown in Fig. 8A, the levels of ATP production in mutant cells in the presence of glucose (total cellular levels of ATP) ranged from 77% to 86%, with an average of 82% relative to the mean value measured in the control cell lines (*P* < 0.001). In contrast, as shown in Fig. 8B, the levels of ATP production in mutant cell lines, in the presence of pyruvate and 2-deoxy-d-glucose to inhibit the glycolysis (mitochondrial levels of ATP), ranged from 64% to 77%, with an average of 70% relative to the mean value measured in the control cell lines (*P* = 0.002).

### Decrease of mitochondrial membrane potential.

**FIG 9:**
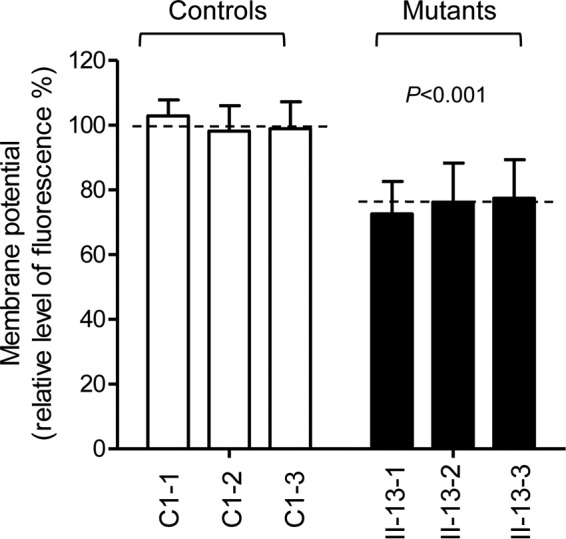
Mitochondrial membrane potential analysis. The mitochondrial membrane potential (ΔΨm) was measured in three mutant and three control cell lines using a JC-10 fluorescent probe assay system. The ratios of fluorescence intensities at Ex/Em wavelengths of 490/590 nm and 490/530 nm (FL_590_/FL_530_) were recorded to delineate the ΔΨm level of each sample. The relative ratios of the FL_590_/FL_530_ geometric means between mutant and control cell lines were calculated to reflect the level of ΔΨm. The average of three to five determinations for each cell line is shown. Graph details and symbols are explained in the legend to Fig. 3.

The mitochondrial membrane potential (ΔΨm) is the central bioenergetic parameter that controls respiratory rate, ATP synthesis, and the generation of ROS and is itself controlled by electron transport and proton leaks. The levels of ΔΨm were measured in three mutant and three control cell lines using a JC-10 fluorescent probe assay system. The ratios of fluorescence intensity at excitation/emission (Ex/Em) wavelengths of 490/590 and 490/530 nm (FL_590_/FL_530_) were recorded to determine the ΔΨm level of each sample. The relative ratios of the FL_590_/FL_530_ geometric mean between mutant and control cell lines were calculated to represent the level of ΔΨm. As shown in Fig. 9, the levels of the ΔΨm in the mutant cell lines carrying the m.5655A → G mutation ranged from 72.6 to 77.4%, with an average of 75.4% (*P* < 0.001) of the mean value measured in the control cell lines. In contrast, the levels of ΔΨm in mutant cells in the presence of FCCP [carbonyl cyanide-*p*-(trifluoromethoxy)phenylhydrazone] were comparable to those measured in the control cell lines (data not shown).

### The increase of mitochondrial ROS production.

**FIG 10:**
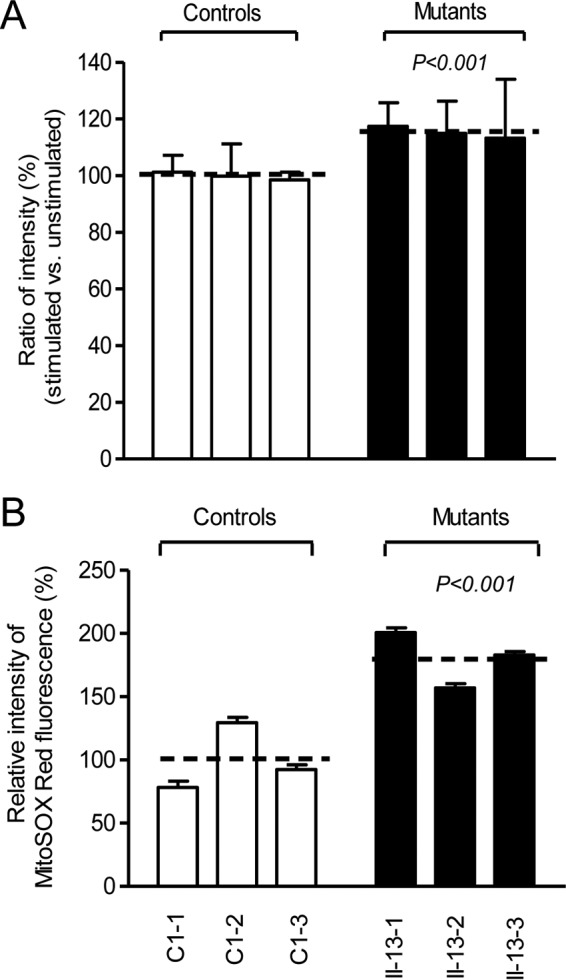
Measurement of cellular and mitochondrial ROS production. (A) Relative levels of ROS production in total cells. The rates of ROS production in total cells from three mutant cell lines and three control cell lines were analyzed by a BD-LSR II flow cytometer system with or without H_2_O_2_ stimulation. The relative ratio of intensity (H_2_O_2_-stimulated versus unstimulated cells) was calculated. (B) Relative levels of ROS production in mitochondria. The levels of ROS generation by mitochondria in living cells from three mutant cell lines and three control cell lines were determined using the mitochondrial superoxide indicator MitoSOX-Red. Fluorescence was measured using a FACSCalibur instrument (BD Biosciences), with excitation at 488 nm and emission at 580 nm. The data were analyzed with FlowJo software. The average of three determinations for each cell line is shown. Graph details and symbols are explained in the legend to Fig. 3.

The total cellular levels of ROS generation in the vital cells derived from three mutant cell lines carrying the m.5655A → G mutation and three control cell lines lacking the mutation were measured with flow cytometry under normal conditions and under H_2_O_2_ stimulation (32, 40, 43). Geometric mean intensity was recorded to measure the rate of ROS of each sample. The ratio of geometric mean intensity between unstimulated and H_2_O_2_-stimulated cells was calculated to delineate the reaction upon increasing levels of ROS under oxidative stress. As shown in Fig. 10A, the total cellular levels of ROS generation in the mutant cell lines carrying the m.5655A → G mutation varied from 103% to 115%, with an average of 110% (*P* = 0.001) of the mean value measured in the control cell lines. Furthermore, the levels of mitochondrial ROS (mitoROS) among the cybrids were determined using a MitoSOX assay via flow cytometry (40, 41). As shown in Fig. 10B, the levels of mitochondrial ROS generation in the mutant cell lines carrying the m.5655A → G mutation ranged from 157% to 201%, with an average of 180% (*P* = 0.001) of the mean value measured in the control cell lines.

## DISCUSSION

In the present study, we investigated the pathogenic mechanism of the hypertension-associated m.5655A → G mutation in the mitochondrial tRNA^Ala^ gene. The occurrence of the m.5655A → G mutation in three genetically unrelated Chinese families affected by hypertension and differing considerably in their mtDNA haplotypes strongly indicated that this mutation is involved in the pathogenesis of this disorder. In fact, the m.5655A → G mutation was located at the 5′ end of tRNA, which is the processing site for the tRNA^Ala^ 5′ end precursors of the light strand, catalyzed by RNase P (15, 19, 22). It was hypothesized that the m.5655A → G mutation caused the structural and functional alterations in the tRNA^Ala^. The observation that the m.5655A → G mutation caused an ∼35% reduction in the efficiency of the 5′ end processing of tRNA^Ala^ precursor indicated that the primary defect arising from the m.5655A → G mutation was the perturbed processing of the tRNA^Ala^ 5′ end precursor. The mutation is also localized at a highly conserved nucleotide (1A) to form a base pairing (1A-72U) on the acceptor stem of the tRNA^Ala^ (17, 44). This nucleotide may play an important role in the stability and identity of tRNA (17, 21). The single 1A-72U base pairing of mitochondrial tRNA^Ala^ is an important recognition site for its cognate aminoacyl-tRNA synthetase (45, 46). The m.5655A → G mutation led to the improperly aminoacylated tRNA^Ala^, as suggested by the aberrantly aminoacylated tRNA^Ala^ in the mutant cell lines and the slower electrophoretic mobility of mutated tRNA with respect to the wild-type molecules, as in the cases of the tRNA^Thr^ 15927A → G and tRNA^His^ 12201T → C mutations (32, 47, 48). An improperly aminoacylated tRNA then makes the mutant tRNA^Ala^ metabolically less stable and more subject to degradation, thereby lowering the level of the tRNA (15, 36, 49). Thus, both the altered processing of the tRNA^Ala^ 5′ end precursor and the improperly aminoacylated tRNA contributed to the lower level of tRNA^Ala^ in cybrid cell lines carrying the m.5655A → G mutation. However, the 43% reduction in the steady-state level of mutant tRNA^Ala^ in cybrids carrying the m.5655A → G mutation was above the proposed threshold level (70% reduction) to produce a clinical phenotype (9, 32, 33). Therefore, the m.5655A → G mutation is the primary causative factor but is itself insufficient to produce the hypertension phenotype, as in the case of the hypertension-associated m.4263A → G mutation (15).

A shortage and aberrant aminoacylation of mutant tRNA^Ala^ should be responsible for the impairment of mitochondrial translation and inefficient oxidative phosphorylation. In fact, the mtDNA encoded 13 polypeptides in the complexes of the oxidative phosphorylation system (ND1 to ND6 and ND4L of complex I; CYTB of complex III; CO1, CO2, and CO3 of complex IV; and ATP6 and ATP8 of complex V) (7). In the present study, the reduced levels of mitochondrial proteins (an average decrease of ∼29%) were comparable with the reduced rate of mitochondrial protein synthesis observed in cell lines carrying the hypertension-associated m.4435A → G and m.4263A → G mutations (14, 15). There were variable decreases in levels of six mtDNA-encoded polypeptides in the mutant cell lines. Notably, marked reductions in the levels of ND1 and ATP6 with a high proportion of alanine codons were observed in mutant cell lines, while only mild reductions occurred in the levels of the other four polypeptides with relatively lower proportions of alanine codons in the mutant cell lines. As shown in Table 1, polypeptide levels in mutant cell lines, relative to those in control cell lines, did not significantly correlate with either the number or proportion of alanine codons. The impairment of mitochondrial translation was apparently responsible for the reduced activities of the mitochondrial respiration chain, especially marked reductions in basal OCR, ATP-linked OCR, or maximal OCR in mutant cells. In particular, the impaired synthesis of ND1 may be the major contributor to the decreased activity of complex I, while the mildly reduced synthesis of CYTB and CO2 may alter the activities of complexes III and IV. This suggested that the activity of complex I is the rate-limiting step (50). The respiratory deficiency caused by the m.5655A → G mutation may result in uncoupling of the oxidative pathway for ATP synthesis, oxidative stress, and subsequent failure of cellular energetic process (51). In this study, a 30% reduction in mitochondrial ATP production in cybrids carrying the m.5655A → G mutation may result from the reduced activities of complexes I and IV as well as from the defective synthesis of ATP6 polypeptide. *In vivo*, skeletal and vascular smooth muscles carrying the m.5655A → G mutation may be particularly sensitive to increased ATP demand (4). Furthermore, the decreased efficiency of respiration by the tRNA mutations often alters mitochondrial membrane potential (32, 52). In this study, a 25% reduction in mitochondrial membrane potential was observed in mutant cell lines carrying the m.5655A → G mutation, in contrast to the marked reductions in cells carrying the 12201T → C mutation (32).

In fact, mitochondrial respiration is the major source of ROS, with 0.2% of oxygen consumed being normally converted into reactive oxygen species in a quiescent state (53). Impairment of electron flow in the electron transport chains (ETC) due to mutations affecting one of the complexes (I, II, and III) might lead to electrons escaping to oxygen at an upstream site (50, 54). In this study, the decreased activity of complex I caused by the 5655A → G mutation may be the major contributor to the increasing production of ROS at total cellular and mitochondrial levels. Unlike mild elevation of total cellular ROS production, an 80% increase in mitochondrial ROS generation in the cells carrying the m.5655A → G mutation indicated that the defective mitochondria are the major producers of ROS (54). In turn, the increased levels of cytosolic ROS may produce damage to mitochondrial proteins, nucleic acids, and lipids, stimulating a forward feeding loop of mitochondrial ROS generation and aggravated cell damage (55, 56). The skeletal and vascular smooth muscles may be preferentially involved because they were somehow exquisitely sensitive to inefficient metabolism, a subtle imbalance in cellular redox state, or an increased level of free radicals (4, 54, 56). An inefficient metabolism caused by the mitochondrial dysfunction in skeletal and vascular smooth muscles may lead to the elevation of systolic blood pressure and therefore be involved in hypertension. The homoplasmic form, mild mitochondrial dysfunction, late onset, and incomplete penetrance of hypertension observed in these Chinese families carrying the m.5655A → G mutation suggest that the mutation is an inherited risk factor necessary for the development of hypertension but may by itself be insufficient to produce a clinical phenotype. The nuclear genetic or epigenetic factors may contribute to the development of clinical phenotype in subjects carrying the m.5666A → G mutation (57, 58). In particular, the tissue-specific effect of this tRNA mutation may be attributed to the tissue-specific RNA metabolism or the involvement of nuclear modifier genes (59).

In summary, our findings suggest the pathogenic mechanism leading to an impaired oxidative phosphorylation in cybrid cell lines carrying the hypertension-associated tRNA^Ala^ 5655A → G mutation. The m.5655A → G mutation alters the secondary structure and function of tRNA. A failure in tRNA metabolism impaired mitochondrial translation and respiration. As a result, this respiratory deficiency reduced mitochondrial ATP production, increased the production of oxidative reactive species, and subsequently elevated blood pressure. Thus, our findings may provide new insights into the understanding of the pathophysiology of maternally inherited hypertension.

## Supplementary Material

Supplemental material
