# Supplementary material for: A Hypertension-Associated tRNAAla Mutation Alters tRNA Metabolism and Mitochondrial Function
Source: Mol Cell Biol. 2016 Jun 29;36(14):1920–30. doi: 10.1128/MCB.00199-16 (PMC4936059; doi:10.1128/MCB.00199-16)
Supplement: Supplemental material [file supp_36_14_1920__index.html]

Supplemental material 

# A Hypertension-Associated tRNAAla Mutation Alters tRNA Metabolism and Mitochondrial Function

## Supplemental material

- Supplemental file 1 -

  Tables S1 (mtDNA variants in three Han Chinese probands) and S2 (Clinical data for some members in three Chinese pedigrees)

  PDF, 42K
