# Supplementary material for: A Hypertension-Associated tRNAAla Mutation Alters tRNA Metabolism and Mitochondrial Function
Source: Mol Cell Biol. 2016 Jun 29;36(14):1920–30. doi: 10.1128/MCB.00199-16 (PMC4936059; doi:10.1128/MCB.00199-16)
Supplement: Supplemental material [file MCB.00199-16_zmb999101258so1.pdf]

Table S1. mtDNA variants in three Han Chinese probands with hypertension and one control subject (C1)

Gene	Position	Replacement	Conservation Index [a] (17 species)	Frequency [b]	CRS	WHP20-II-13	WHP21-II-3	WHP22-II-5	C1	Previously reported [b]
D-loop	73	A to G			A	G	G	G	G	Yes
	150	C to T			C				T	Yes
	152	T to C			T		C			Yes
	195	T to C			T	G				Yes
	263	A to G			A	G	G	G	G	Yes
	298	C to T			C		T	T		Yes
	310	T to TC/TCC			T	TC	TCC	TCC	TC	Yes
	316	T to TC			T	TC	TC	TC		Yes
	489	T to C			T		C	C		Yes
	16093	T to C			T	C				Yes
	16136	T to C			T				C	Yes
	16140	T to C			T				C	Yes
	16150	C to T			C	T				Yes
	16169	C to T			C		T	T		Yes
	16182	A to C			A	C				Yes
	16183	A to C			A	C			C	Yes
	16189	T to C			T	C			C	Yes
	16193	C to CC			C				CC	Yes
	16217	T to C			T	C			C	Yes
	16223	C to T			C		T	T		Yes
	16240	A to G			A	G				Yes

	16249	T to C			T				C	Yes
	16261	C to T			C	T				Yes
	16274	G to A			G		A	A	A	Yes
	16280	A to G			A				G	Yes
	16291	C to T			C				T	Yes
	16311	T to C			T		C	C		Yes
	16362	T to C			T		C	C		Yes
	16407	C to T			C	C				Yes
	16519	T to C			T				C	Yes
12S rRNA	709	G to A	10/17	444/2704	G				A	Yes
	750	A to G	17/17	2682/2704	A	G	G	G	G	Yes
	1119	T to C	9/17	26/2704	T				C	Yes
	1438	A to G	16/17	2620/2704	A	G	G	G	G	Yes
	1556	C to T	4/17	0/2704	C	T				Yes
16S rRNA	1842	A to G	3/17	5/2704	A	G				Yes
	2483	T to C	6/17	7/2704	T				C	Yes
	2706	A to G	15/17	2178/2704	A	G			G	Yes
	3010	G to A	6/17	550/2704	G		A	A		Yes
ND1	3435	C to T			C				T	No
	3497	C to T (Ala64Val)	3/17	22/2704	C				T	Yes
	3571	C to T (Leu89Phe)	16/17	2/2704	C				T	No
	4140	C to T			C		T	T		No
$tRNA^{Gln}$	4394	C to T	9/17	3/2704	C			T		Yes
ND2	4769	A to G			A		G	G		Yes

	4883	C to T			C		T	T		Yes
	5178	C to T (Leu237Met)	8/17	1/2704	C		A	A		Yes
**tRNA^{Ala}**	**5655**	T to C	10/17	23/2704	**T**	**C**	**C**	**C**		Yes
COXI	6286	T to C (Val128Ala)	17/17	1/2704	T	C				Yes
	6863	A to G			A	G				Yes
	7028	C to T			C	T	T	T	T	Yes
NC7	8281-8289	9bp del			C	9bp del			9 bp del	Yes
ATP8	8414	C to T (Leu17Phe)	10/17	264/2704	C		T	T		Yes
	8519	G to A (Glu52Lys)	12/17	1/2704	G		A	A		Yes
ATP6	8701	A to G (Thr59Ala)	9/17	933/2704	A		G	G		Yes
	8860	A to G (Thr112Ala)	12/17	2698/2704	A	G			G	Yes
ATP6	9128	T to C (Ile201Thr)		6/2704	T				C	Yes
CO3	9368	A to G			A		G	C		Yes
	9540	T to C			T		C	C		Yes
	9575	G to A			G				A	Yes
ND3	10398	A to G (Thr114Ala)	7/17	1242/2704	A	G	G	G		Yes
	10400	C to T (Thr114Ala)	7/17	724/2704	C		T	T		Yes
ND4L	10493	T to C			T				C	No
	10873	T to C			T		C	C		Yes
	11059	C to T			C		T	T		Yes
ND4	11440	G to A			G				A	Yes
	11719	G to A			G	A	A	A	A	Yes
ND5	12372	G to A			G		A	A		Yes
	12705	C to T			C		T	T		Yes
	13104	A to G			A		G	G		Yes
	13135	G to A (Ala267Ile)	4/17	18/2704	G	A				Yes

ND6	14410	G to A			G	A				Yes
	14668	C to T			C		T	T		Yes
Cytb	14766	C to T (Thr7Ile)	8/17	610/2704	C	T		T	T	Yes
	14783	T to C			T		C	C		Yes
	15043	G to A			G		A	A		Yes
	15085	A to G			A		G	G		Yes
	15301	G to A			G		A	A		Yes
	15326	A to G (Thr194Ala)	9/17	2687/2704	A	G	G	G	G	Yes
tRNA^{Thr}	15924	T to C	14/17	106/2704	T			C		Yes

CRS, consensus Cambridge sequence (Andrews et al., 1999)

[a] Conservation index (CI) was calculated by comparing the human mtDNA variants with other 16 vertebrates: *Bos Taurus, Cebus albifrons, Gorilla gorilla, Hylobates lar, Lemur catta, Macaca mulatta, Macaca sylvanus, Mus musculus, Nycticebus coucang, Pan paniscus, Pan troglodytes, Pongo pygmaeus, Pongo abelii, Papio hamadryas, Tarsius bancanus,and Xenopus laevis*.

[b] See the online mitochondrial genome database http://www.mitomap.org/MITOMAP and http://www.mtdb.igp.uu.se/.

Table S2. Summary of Clinical Data for Some Members in three Chinese Pedigree

Subjects	Gender	Age of test (yrs)	Age of onset (yrs)	Diastolic pressure (mmHg)	Diastolic pressure (mmHg)
WHP20-I-2	F	94	80	180	90
WHP20-II-2	F	73	50	160	90
WHP20-II-3	M	69	64	170	80
WHP20-II-5	M	66	60	140	100
WHP20-II-6	F	62	48	150	110
WHP20-II-7	M	63	62	142	90
WHP20-II-9	M	60	55	160	110
WHP20-II-12	F	57	56	140	110
WHP20-II-13	M	55	50	210	130
WHP20-II-12	M	56	53	155	95
WHP20-III-1	M	55	40	180	110
WHP20-III-2	F	45	46	145	85
WHP20-III-3	M	52	52	140	90
WHP20-III-4	F	52	49	180	110
WHP20-III-6	F	48	42	170	110
WHP20-III-8	F	45	44	170	110
WHP20-III-20	M	40	-	110	70
WHP20-III-21	M	34	-	118	76
WHP20-IV-2	M	39	38	145	90

WHP20-IV-3	F	35	-	110	75
WHP20-IV-4	F	33	-	110	72
WHP20-IV-5	M	31	-	105	76
WHP20-IIV-6	F	28	-	114	70
WHP20-IIV-7	M	26	-	110	75
WHP21-I-2	F	75	-	135	78
WHP21-II-1	M	56	-	130	83
WHP21-II-3	F	55	55	145	98
WHP21-II-3	M	54	52	150	95
WHP21-II-8	F	48	-	125	79
WHP21-III-3	M	45	44	148	95
WHP21-III-4	M	44	-	120	75
WHP21-III-6	F	28	-	115	76
WHP21-III-7	F	26	-	112	70
WHP22-I-2	F	89	69	153	90
WHP22-II-1	F	69	64	165	110
WHP22-II-3	F	67	62	170	105
WHP22-II-5	F	65	60	158	101
WHP22-II-7	F	64	-	135	79
WHP22-II-9	M	63	59	156	115
WHP22-III-1	M	52	52	152	101
WHP22-III-2	M	51	-	135	79

WHP22-III-3	M	50	50	146	110
WHP22-III-4	F	49	-	138	84
WHP22-III-5	F	50	49	140	95
WHP22-III-6	F	48	-	138	82
WHP22-III-7	M	46	-	136	85
WHP22-III-8	F	48	-	130	79
WHP22-III-9	M	45	44	149	98
WHP22-III-10	M	44	-	130	76
WHP22-III-11	F	40	-	136	86

These patients had antihypertension treatment. This table shows pre-treatment blood pressures.
